# dNP2 is a blood–brain barrier-permeable peptide enabling ctCTLA-4 protein delivery to ameliorate experimental autoimmune encephalomyelitis

**DOI:** 10.1038/ncomms9244

**Published:** 2015-09-15

**Authors:** Sangho Lim, Won-Ju Kim, Yeon-Ho Kim, Sohee Lee, Ja-Hyun Koo, Jung-Ah Lee, Heeseok Yoon, Do-Hyun Kim, Hong-Jai Park, Hye-Mi Kim, Hong-Gyun Lee, Ji Yun Kim, Jae-Ung Lee, Jae Hun Shin, Lark Kyun Kim, Junsang Doh, Hongtae Kim, Sang-Kyou Lee, Alfred L. M. Bothwell, Minah Suh, Je-Min Choi

**Affiliations:** 1 Department of Life Science, College of Natural Sciences, Hanyang University, Seoul 133-791, Republic of Korea; 2 Research Institute for Natural Sciences, Hanyang University, Seoul 133-791, Republic of Korea; 3 Center for Neuroscience Imaging Research (CNIR), Institute for Basic Science (IBS), Suwon 440-746, Republic of Korea; 4 Samsung Advanced Institute for Health Sciences & Technology (SAIHST), Seoul 135-710, Republic of Korea; 5 Division of Integrative Bioscience and Biotechnology, Pohang University of Science and Technology, Pohang 790-784, Republic of Korea; 6 Department of Immunobiology, Yale University School of Medicine, New Haven, Connecticut 06520, USA; 7 Department of Mechanical Engineering, School of Interdisciplinary Bioscience and Bioengineering, Pohang University of Science and Technology, Pohang 790-784, Republic of Korea; 8 Department of Biological Science, Sungkyunkwan University, Suwon 440-746, Republic of Korea; 9 Department of Biotechnology, Yonsei University, Seoul 120-749, Republic of Korea; 10 Department of Biomedical Engineering, Sungkyunkwan University, Suwon 440-746, Republic of Korea

## Abstract

Central nervous system (CNS)-infiltrating effector T cells play critical roles in the development and progression of multiple sclerosis (MS). However, current drugs for MS are very limited due to the difficulty of delivering drugs into the CNS. Here we identify a cell-permeable peptide, dNP2, which efficiently delivers proteins into mouse and human T cells, as well as various tissues. Moreover, it enters the brain tissue and resident cells through blood vessels by penetrating the tightly organized blood–brain barrier. The dNP2-conjugated cytoplasmic domain of cytotoxic T-lymphocyte antigen 4 (dNP2-ctCTLA-4) negatively regulates activated T cells and shows inhibitory effects on experimental autoimmune encephalomyelitis in both preventive and therapeutic mouse models, resulting in the reduction of demyelination and CNS-infiltrating T helper 1 and T helper 17 cells. Thus, this study demonstrates that dNP2 is a blood–brain barrier-permeable peptide and dNP2-ctCTLA-4 could be an effective agent for treating CNS inflammatory diseases such as MS.

## Abstract (web summary)

Most of the cell penetrating peptides can transport therapeutic agents across plasma membranes but barely across the blood-brain barrier. Here the authors develop a peptide that can enter the brain, and show that its fusion to immunomodulatory protein ctCTLA-4 is effective in a mouse model of multiple sclerosis.

## Graphical abstract

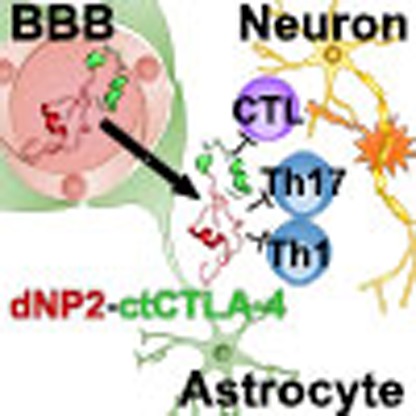

Multiple sclerosis is a human autoimmune disease caused by the induction of inflammation in the central nervous system (CNS) by myelin-specific T cells that cross the protective environment of the blood–brain barrier (BBB)^1^. The mouse model of multiple sclerosis, experimental autoimmune encephalomyelitis (EAE), induced by interferon-γ (IFN-γ) or interleukin-17A (IL-17A) expressing T helper 1 (Th1) or T helper 17 (Th17) cells following MOG_35–55_ immunization, has been intensively studied^2^,^3^. Th1 and Th17 cells infiltrate the CNS, including the brain and spinal cord, and in high levels of these cells induce inflammation and nerve cell apoptosis^3^,^4^.

Although these effector cells could be potent therapeutic targets, the BBB and the blood–spinal cord barrier (BSCB) greatly restrict the delivery of therapeutic biomolecules into the CNS^5^,^6^. Various strategies have been developed to overcome the restrictions on drug delivery imposed by the BBB/BSCB^7^. Opening the barrier by stimulating tight junctions in the BBB/BSCB, through the use of biological, chemical or physical stimuli, has been the most widely used method of drug delivery. For example, Cereport, a synthetic peptide that can disrupt tight junctions, has been widely studied for BBB permeability and therapeutic approaches^8^,^9^. The use of high-intensity-focused ultrasound has also been investigated to physically enhance drug delivery into the CNS for therapeutic purposes^10^. Another strategy uses cationic cell-permeable peptides (CPP) via adsorptive-mediated transcytosis of barrier cells. For instance, the most well-known CPP, TAT, was fused with the anti-apoptotic protein B-cell lymphoma-extra large (Bcl-xl) and delivered to the murine brain as a treatment for ischaemic brain injury^11^,^12^. Other CPPs, such as SynB1/3, have also been used to deliver drugs across the BBB. SynB1-conjugated doxorubicin or penicillin and SynB3-conjugated dalargin exhibit significantly increased brain uptake^13^,^14^,^15^.

However, because all of the above methods originate from synthetic chemicals or other organisms and may have long-term toxicity or other side effects, which limit their clinical applications in humans, further improvements are needed to obtain a safe, efficient system for drug delivery into the CNS. In the current study, we identified and optimized a human-derived CNS-permeable CPP and applied it to an EAE model via conjugation with the cytoplasmic domain of cytotoxic T-lymphocyte antigen 4 (ctCTLA-4) to control autoimmune effector T-cell responses in the CNS.

CTLA-4 is an immune regulatory receptor expressed on the surface of T cells and interacts with the B7 molecules expressed on antigen-presenting cells, thereby competing with CD28 and transmitting negative signals to T cells^16^,^17^,^18^,^19^. The *Ctla-4* gene consists of four exons, which encode the leader sequence (exon 1), the extracellular ligand-binding domain (exon 2), the transmembrane region (exon 3) and the cytoplasmic tail (exon 4). Alternative splicing of the *Ctla-4* gene results in the expression of various CTLA-4 isoforms, including full-length CTLA-4, soluble CTLA-4, ligand-independent CTLA-4 (liCTLA-4) and exon 1/4 CTLA-4 (1/4CTLA-4)^20^. The importance of liCTLA-4, which lacks the ligand-binding domain, in negative immune regulation was demonstrated in murine T cells^21^. In primary T cells, the liCTLA-4 protein inhibits T-cell activation by dephosphorylating the T-cell receptor (TcR) ζ-chain. These studies revealed that the cytoplasmic tail of CTLA-4 could deliver negative signals without B7 binding to the extracellular ligand-binding domain. We also previously reported that intracellular delivery of a recombinant CTLA-4 cytoplasmic domain (ctCTLA-4) efficiently inhibited phosphorylation of the TcR ζ-chain and mitogen-activated protein kinase, resulting in amelioration of allergic airway inflammation and autoimmune arthritis in experimental animal models^22^,^23^.

Here we initially identify a novel CPP, dNP2, from the human novel LZAP-binding protein (NLBP). This peptide exhibits potent protein delivery efficiency in primary mouse and human T cells *in vitro* and even in brain tissue, as determined *in vivo* using live multi-photon confocal microscopy. We then purify recombinant dNP2-conjugated ctCTLA-4 protein, which displays a significant suppressive effect on the activated T cell responses, and we further elucidate its preventive and therapeutic effects on MOG_35–55_-induced autoimmune encephalomyelitis in mice.

## Results

### Identification and optimization of a dNP2

**Figure 1:**
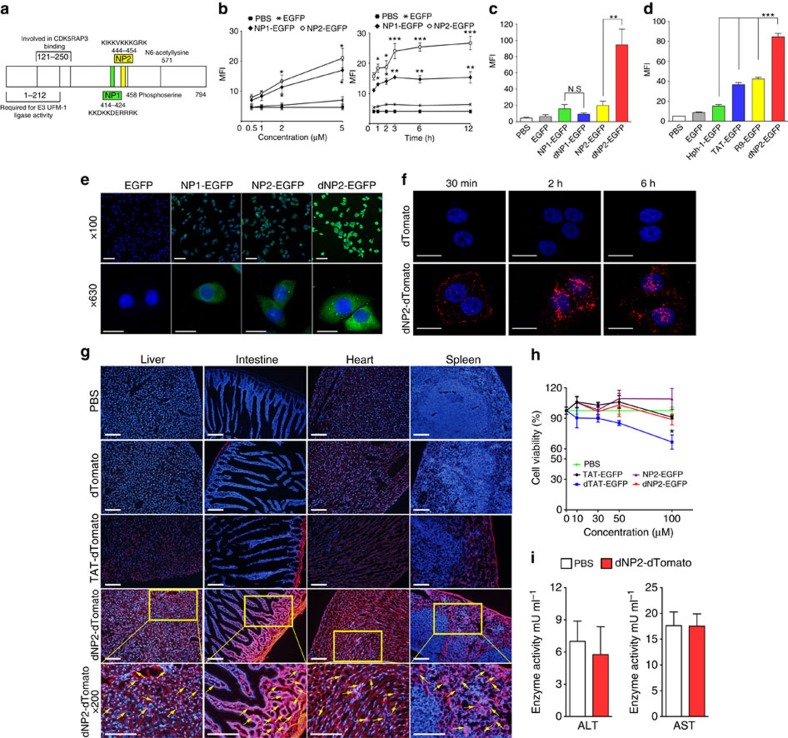
Identification and optimization of a human-derived cell-permeable peptide, dNP2. (**a**) The NP1 and NP2 sequences are located in a central domain of human NLBP. (**b**) Jurkat T cells were incubated with various concentrations (0.5–5 μM) of EGFP, NP1- and NP2-EGFP for 2 h and various time periods (0.5–12 h) with 5 μM protein. Intracellular fluorescence was analysed by flow cytometry and is represented as mean fluorescence intensity (MFI) of the cells. (**c**) Jurkat T cells were incubated with 5 μM EGFP, NP1-, dNP1-, NP2-, dNP2-EGFP or PBS for 2 h and the data were analysed as described above. (**d**) Jurkat T cells were incubated with 5 μM EGFP, Hph-1-, TAT-, R9-, dNP2-EGFP or PBS for 2 h and the data were analysed described above. (**e**) HeLa cells were visualized after a 2 h incubation with EGFP, NP1- NP2 or dNP2-EGFP (20 μM) using a DeltaVision system at × 100 (Scale bar, 100 μm) or × 500 (Scale bar, 20 μm) magnification. (**f**) The nuclear localization of the dNP2-dTomato in HeLa cells was observed after a 30 min, 2 or 6 h incubation with the proteins using a confocal microscope at × 400 (Scale bar, 20 μm) magnification. (**g**) Five milligrams dTomato, TAT- or dNP2-dTomato was intraperitoneally injected into 7-week-old female C57BL/6 mice. At 2 h after injection, the tissues were harvested and prepared as frozen slides. The nuclei were stained with Hoechst and fluorescence was observed via fluorescence microscopy. Yellow boxes in the images indicate magnified region ( × 100; Scale bar, 200 μm, × 200; Scale bar, 100 μm). (**h**) HeLa cells were incubated with various concentrations (10, 30, 50 or 100 μM) of TAT-, dTAT-, NP2-, dNP2-EGFP or PBS for 24 h. Cell viability was analysed by water-soluble tetrazolium-8 based cell counting kit-8 (CCK-8). (**i**) Alanine aminotransferase (ALT) activity and aspartate aminotransferase (AST) activity in serum of every other day injected mice by 5 mg kg^−1^ dNP2-dTomato, dNP2-ctCTLA-4 or PBS for 14 days were measured using an ALT/AST activity assay kit. Values are mean±s.e.m. and *P<0.05; **P<0.01; ***P<0.001; Student's *t*-test.

To identify a novel human origin CPP, we performed homology searches against the human proteome database using previously reported CPP sequence information. We identified candidate CPP sequences from the human NLBP, which consists of 794 amino acids and contains two functional regions: residues 1–212, which are required for E3 UFM-1 ligase activity, and residues 121–250, which are involved in CDK5RAP3-binding activity^24^,^25^. On the basis of BLAST homology analysis, two candidate sequences, which are highly conserved among various species, were selected: NP1 (414–424; KKDKKDERRRK) and NP2 (444–454; KIKKVKKKGRK; Fig. 1a, Supplementary Fig. 1). To examine the intracellular protein delivery activity of these CPP candidates, we initially constructed plasmids expressing enhanced green fluorescent proteins (EGFPs), including NP1-EGFP, NP2-EGFP, and EGFP without a CPP, and then purified the resulting proteins (Supplementary Fig. 2), as previously described^26^. These proteins were examined by incubation with cultured cells to determine their intracellular transduction efficiency. Flow cytometric analysis demonstrated that significant transduction of the NP1-EGFP and NP2-EGFP proteins into Jurkat T cells occurred in both concentration- and time-dependent manners (Fig. 1b and Supplementary Fig. 3). We then determined whether the tandem repeats of NP1 or NP2 (dNP1 or dNP2) or a combination of the two candidates (NP1-2 or NP2-1) exhibit higher delivery efficiencies than their monomeric forms. Interestingly, only dNP2-EGFP exhibited dramatically enhanced delivery efficiency; dNP1, NP1-2 and NP2-1 did not exhibit any significant enhancement of intracellular transduction efficiency compared with the monomeric form (Fig. 1c, Supplementary Fig. 4). The cell-penetrating ability of dNP2 was at least twofold greater than that of TAT or R9 (Fig. 1d), which are well-known CPPs with numerous applications^27^,^28^,^29^. In addition, dNP2-EGFP exhibited efficient intracellular localization into both the cytoplasm and the nucleus (Fig. 1e). To obtain a clearer analysis of nuclear localization, we used a brighter fluorescent protein, dTomato. Substantial intracellular dNP2-dTomato fluorescent signal was detected within 30 min and was clearly localized in the nucleus after 2–6 h (Fig. 1f). Although intracellular delivery of dTAT-EGFP and dHph-1-EGFP was also significantly increased compared with their monomeric forms, dNP2-EGFP exhibited substantially higher efficiency than the other tested proteins (Supplementary Fig. 5a). The enhancement of protein delivery efficiency of dNP2 compared with NP2 was more significant than the protein delivery efficiency ratio of dTAT/TAT or dHph-1/Hph-1 (Supplementary Fig. 5b). Next, we intraperitoneally injected 5 mg of the dTomato, TAT-dTomato or dNP2-dTomato proteins into mice. After 2 h, significant dTomato fluorescence was observed in the various tissues, such as liver, intestine, heart and spleen, of dNP2-dTomato-injected mice and the intensity was brighter and clearer than that of TAT-dTomato (Fig. 1g). On higher magnification ( × 200), dTomato fluorescence was co-localized with nucleus in stained cells in the tissues and we confirmed the *in vivo* intracellular protein delivery efficiency of dNP2 with dNP2-EGFP in the liver ( × 400; Supplementary Fig. 6). As the delivery efficiency of CPP, in general, correlates with cytotoxicity, we performed water-soluble tetrazolium-8 assay to evaluate the *in vitro* cytotoxicity of the proteins. We found no significant toxicity when using up to 100 μM of dNP2-EGFP protein on incubation in HeLa cells while dTAT-EGFP showed ∼70% cell viability at 100 μM (Fig. 1h). To further determine the *in vivo* toxicity, 5 mg kg^−1^ dNP2-dTomato protein was repetitively injected intraperitoneally into mice every other day for 14 days. The mice were then evaluated for signs of *in vivo* toxicity. There were no significant changes in alanine transaminase and aspartate transaminase activity in the serum (Fig. 1i), weight, sizes of the spleen, liver and brain, or apoptotic cells and activated T cells (Supplementary Fig. 7). These results suggest that dNP2 from human NLBP is a potent novel CPP that can deliver functional macromolecules, such as proteins, into cells both *in vitro* and *in vivo* without significant toxicity.

### Efficient protein delivery by dNP2 into primary T cells

**Figure 2:**
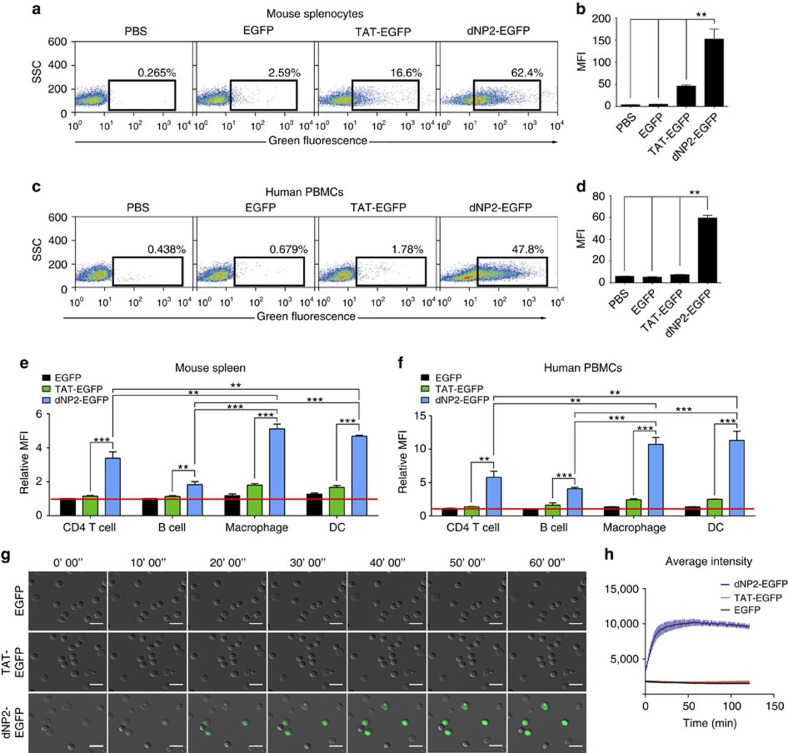
Protein delivery efficiency of dNP2 in primary mouse and human immune cells. (**a**,**b**) Mouse primary splenocytes were isolated from 6-week-old female C57BL/6 mice and the cells were incubated with 5 μM EGFP, TAT- and dNP2-EGFP for 2 h. Intracellular fluorescence was analysed by flow cytometry and the data are represented as dot plots or mean fluorescence intensity (MFI) of the cells. (**c**,**d**) Human PBMCs were isolated from healthy donor blood and the cells were incubated with 5 μM EGFP, TAT-, dNP2-EGFP for 2 h. The data were analysed as described above. (**e**) Total splenocytes were incubated with 1 μM EGFP, TAT-, and dNP2-EGFP for 2 h. Cells were gated using markers specific for CD4 T cells (CD4^+^), B cells (CD19^+^), macrophages (CD11c^lo^CD11b^hi^F480^+^) and DCs (CD11c^hi^MHCII^hi^). The EGFP signal in each cell population was then analysed by flow cytometric analysis. The relative MFI value was normalization to PBS treated cells. The red line indicates relative MFI of PBS-treated cells. (**f**) Total PBMCs were incubated with 1 μM EGFP, TAT-, and dNP2-EGFP for 2 h. Cells were gated with markers specific for CD4 T cells (CD4^+^), B cells (CD19^+^), macrophages (CD11b^+^) and DCs (CD11c^+^) and the data were then analysed as described above. (**g**) Time-lapse images of mouse CD4 T cells incubated with 1 μM EGFP, TAT- and dNP2-EGFP were acquired for 2 h (Scale bar, 15 μm) and (**h**) the average fluorescence intensities of 10 cells from each sample were calculated and plotted. Values are mean±s.e.m. and **P<0.01; ***P<0.001; Student's *t*-test.

Although most CPPs have been evaluated in various cancer cell lines *in vitro*, their penetrating efficiency is generally much lower in primary cells, which severely limits the clinical applications of CPPs in humans^30^. Next, we further examined the delivery efficiency of dNP2 in primary mouse splenocytes and human peripheral blood mononuclear cells (PBMCs). Mouse splenocytes treated with 5 μM dNP2-EGFP displayed 62.4% EGFP-positive cells, while the cells treated with TAT-EGFP or EGFP alone showed 16.6 and 2.59% EGFP-positive cells, respectively, thereby demonstrating the superior intracellular protein delivery efficiency of dNP2 in mouse splenocytes (Fig. 2a,b). Similar results were observed in human PBMCs (Fig. 2c,d), suggesting that dNP2 leads to very efficient delivery of proteins in primary hematopoietic cells, while TAT is much less efficient. Interestingly, both the TAT- and dNP2-EGFP proteins were delivered more efficiently into phagocytic cells such as macrophages and dendritic cells than into lymphocytes, while dNP2 still showed significantly better delivery efficiency in CD4 T cells than did the other controls (Fig. 2e,f). To compare and visualize the efficiencies of dNP2 and TAT in primary T cells, we performed live-cell imaging using magnetic-activated cell sorting-purified total CD4 T cells from C57BL/6 mice. Whereas the fluorescence intensities of T cells treated with 1 μM EGFP and TAT-EGFP remained near basal levels throughout the 2 h imaging period, the average fluorescence intensities of dNP2-EGFP-treated T cells increased linearly and reached a plateau within 30 min (Fig. 2g,h), suggesting that dNP2, but not TAT, is suitable for efficient functional protein delivery into primary T cells.

### Intracellular delivery mechanisms of dNP2

**Figure 3:**
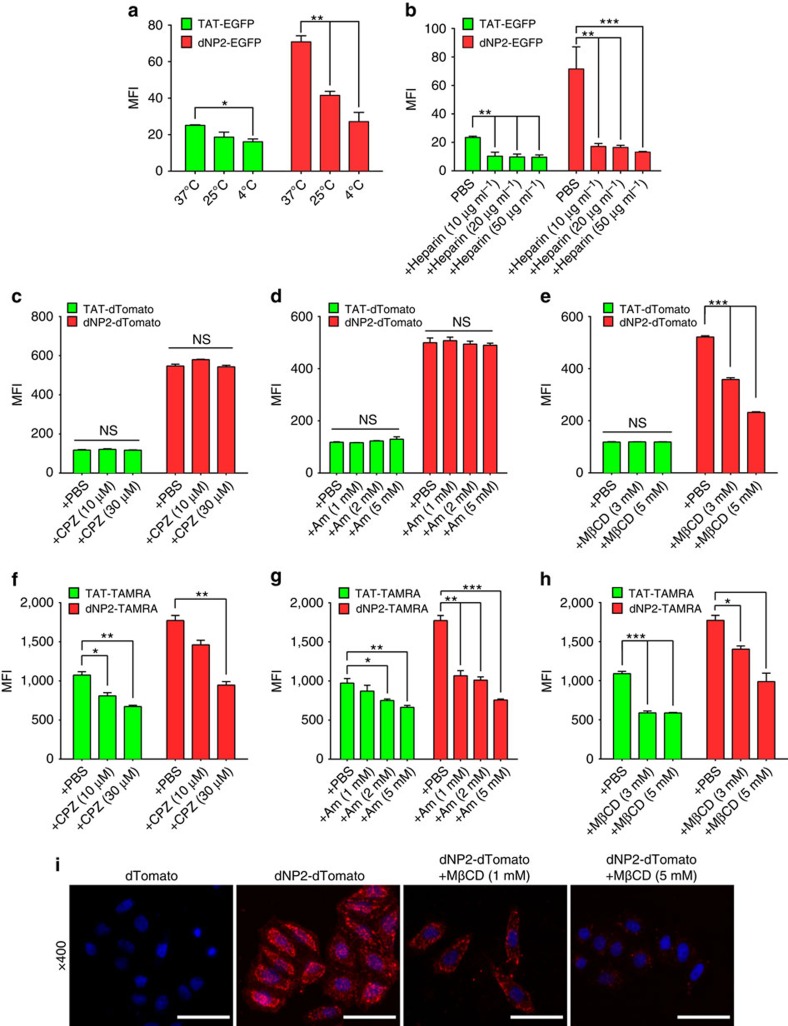
Intracellular delivery mechanisms of dNP2. (**a**) Splenocytes from 6-week-old female C57BL/6 mice were incubated with 5 μM TAT- or dNP2-EGFP at various temperatures (4, 25 or 37 °C) for 2 h. The intracellular EGFP signal of gated CD4 T cells was analysed by flow cytometric analysis and the data are represented as mean fluorescence intensity (MFI). (**b**) The splenocytes were pretreated with 0, 10, 20 or 50 μg ml^−1^ heparin at 37 °C for 30 min and the cells were then further incubated with 5 μM TAT- or dNP2-EGFP at 37 °C for 2 h. The intracellular EGFP protein signal of gated CD4 T cells was analysed and the data are represented as described above. (**c**–**h**) The splenocytes were pre-treated with the indicated concentrations of chlorpromazine (CPZ), amiloride (Am) or methyl-beta cyclodextrin (MβCD) at 37 °C for 30 min and cells were further incubated with (**c**–**e**) 5 μM TAT- or dNP2-dTomato or (**f**–**h**) 5 μM TAMRA-labeled TAT or dNP2 peptide at 37 °C for 1 h. The intracellular dTomato protein signal or TAMRA signal in the CD4 T cells were analysed by flow cytometry. (**i**) HeLa cells were pre-treated with 1–5 mM MβCD or PBS on ice for 10 min and the cells were further incubated with 20 μM dTomato or dNP2-dTomato at 37 °C for 1 h. The intracellular localization of the dTomato protein was visualized by fluorescent microscopy ( × 400, Scale bar, 75 μm). Values are mean±s.e.m. and *P<0.05; **P<0.01; ***P<0.001; Student's *t*-test.

To determine the intracellular delivery mechanisms, we initially examined the delivery efficiency of the dNP2-EGPF protein at different temperatures, including 4, 25 and 37 °C (Fig. 3a). The intracellular delivery efficiencies decreased at lower temperatures, suggesting that delivery may possibly be mediated by energy-dependent mechanisms. We then examined whether the delivery mechanism involves interactions with cell membrane molecules such as glycosaminoglycans, as in the case of the other CPPs^31^,^32^. Heparin, a competitive inhibitor of heparan sulphate on the cell surface, treatment significantly inhibited the delivery efficiency of dNP2 (Fig. 3b), suggesting a requirement for interaction with glycosaminoglycans. Next, we analysed delivery efficiency of dNP2-dTomato protein in mouse CD4 T cells with various endocytosis inhibitors, including chlorpromazine (clathrin-mediated endocytosis inhibitor)^33^, Amiloride (macropinocytosis inhibitor)^34^ and methyl-beta cyclodextrin (MβCD, lipid raft-mediated endocytosis inhibitor)^35^, to determine their effect on intracellular delivery. The intracellular delivery efficiency of the dNP2-dTomato protein was significantly decreased only by MβCD treatment (Fig. 3c–e), suggesting that a lipid raft-mediated endocytosis pathway is one of the major mechanisms of intracellular delivery. Because the delivery mechanism of TAT peptide has been reported to also involve clathrin-mediated endocytosis and macropinocytosis^33^, we hypothesized that peptides and proteins might have different intracellular delivery mechanisms. When mouse splenocytes were incubated with 5 μM TAT- or dNP2-TAMRA peptide with or without chlorpromazine, amiloride or MβCD, the intracellular delivery efficiencies on CD4-positive cells of both the TAT- and dNP2-TAMRA peptides were significantly inhibited by all of the inhibitors, suggesting that the dNP2 peptide alone seems to use multiple endocytosis pathways (Fig. 3f–h). In addition, equivalent pattern was found in total splenocytes (Supplementary Fig. 8). Finally we confirmed and visualized intracellular delivery efficiency of dNP2-dTomato protein in HeLa cells was significantly inhibited by MβCD treatment (Fig. 3i). Collectively, our results demonstrate that the intracellular delivery mechanism of dNP2 is identical to that reported for other CPPs, and utilizes various endocytosis pathways depends on cargos, including lipid raft-mediated endocytosis following heparan sulphate interaction.

### dNP2 delivers cargo proteins into the CNS *in vivo*

**Figure 4:**
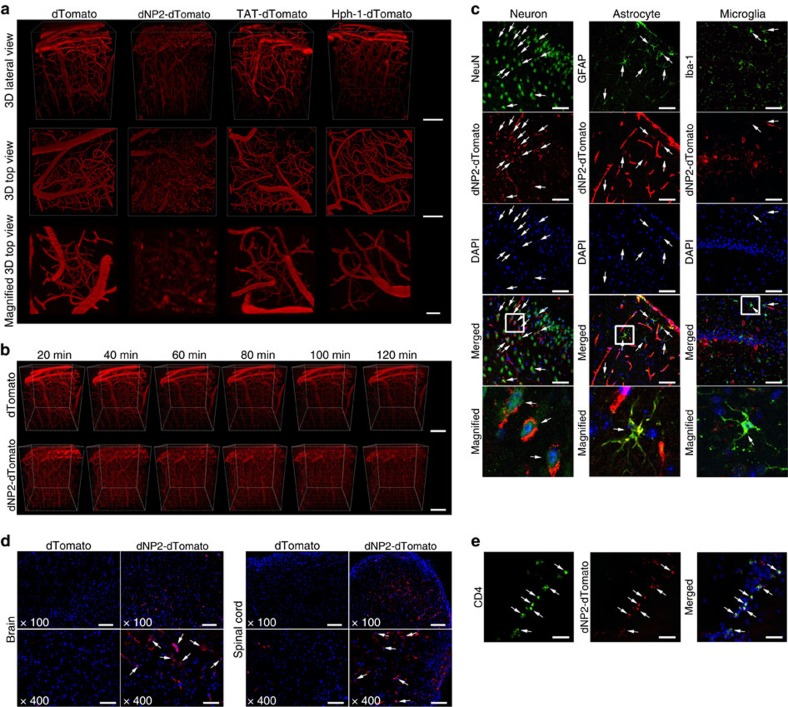
Protein delivery efficiency of dNP2 in brain and spinal cord. 2.5 mg dTomato, Hph-1-, TAT-, and dNP2-dTomato proteins were intravenously injected into 8-week-old male C57BL/6 mice through the tail vein after anaesthesia for real-time live confocal microscopic analysis. (**a**) Mouse brains were observed at 40 min after injection via multi-photon confocal microscopy using a 3D lateral view and 3D top view or magnified 3D top view (Scale bar, 100 μm). (**b**) The fluorescent diffusion of the dNP2-dTomato protein out of blood vessels in the brain was monitored from 20–120 min (Scale bar, 50 μm). (**c**) Co-localization of the dNP2-dTomato (red) signal with fluorescent signals specific for various cell types, including neurons (NeuN, green), astrocytes (GFAP, green), and microglia (Iba-1, green), in frozen sectioned brain tissue following confocal microscopy ( × 400, Scale bar, 50 μm) is indicated by white arrows. White boxes in the merged images are magnified regions. (**d**–**e**) EAE was induced in mice as described in the Materials and Method section, 2.5 mg of the dTomato or dNP2-dTomato proteins were subsequently injected intravenously. After 1 h, the brain and spinal cord tissues were harvested and prepared as frozen slides. (**d**) The cellular localization of the dNP2-dTomato protein in the brain or spinal cord tissues was visualized by fluorescence microscopy ( × 100; Scale bar, 200 μm, × 400; Scale bar, 50 μm). (**e**) The co-localization of the dNP2-dTomato protein with CD4-positive cells (green) was analysed by fluorescence microscopy ( × 400, Scale bar, 50 μm) and is indicated as white arrows.

The high protein-delivery efficiency of dNP2 in primary T cells, which are generally the most difficult of cells to transfect, prompted us to investigate the membrane-penetrating efficiency of dNP2 in brain. To visualize the CNS-permeable ability of dNP2, we performed real-time *in vivo* multi-photon confocal imaging of mouse brains after intravenous injection of 2.5 mg dTomato, TAT-dTomato, Hph-1-dTomato or dNP2-dTomato proteins. Although each injection allowed the successful visualization of the vasculature of the intact mouse brain via red fluorescence after 40 min, the red fluorescence signal diffused out of the vasculature and into cellular structures only in animals injected with dNP2-dTomato (Fig. 4a). In the case of TAT- and Hph-1-dTomato injections, significant diffusion into cellular structures was not observed; however, the diffusion of dNP2-dTomato fluorescence out of blood vessels increased in a time-dependent manner up to 120 min after injection (Fig. 4b). Using frozen brain tissue sections, staining for various cell-type markers revealed that the dNP2-dTomato protein signal was significantly co-localized with neurons (Neuronal Nuclei (NeuN)-positive cells), astrocytes (Glial fibrillary acidic protein (GFAP)-positive cells) and microglial cells (Ionized calcium binding adaptor molecule 1 (Iba-1)-positive cells), suggesting that the dNP2-dTomato protein was successfully delivered into brain cells through the blood vessels (Fig. 4c). Next, we confirmed the intracellular delivery efficiency of dNP2-dTomato in the brain and spinal cord tissues in MOG_33–55_-immunized inflammatory EAE mice. The dNP2-dTomato signal was significantly detected in the inflammatory brain and spinal cord tissues (Fig. 4d), and was also co-localized with CD4-positive cells in the brain (Fig. 4e), suggesting that dNP2 can deliver a protein into infiltrating T cells in the brain. These results suggest that dNP2 can deliver functional proteins into the brain and spinal cord tissues and into resident or migratory cells; thereby suggesting its valuable application potency in CNS inflammatory diseases.

### dNP2-ctCTLA-4 significantly ameliorates EAE

**Figure 5:**
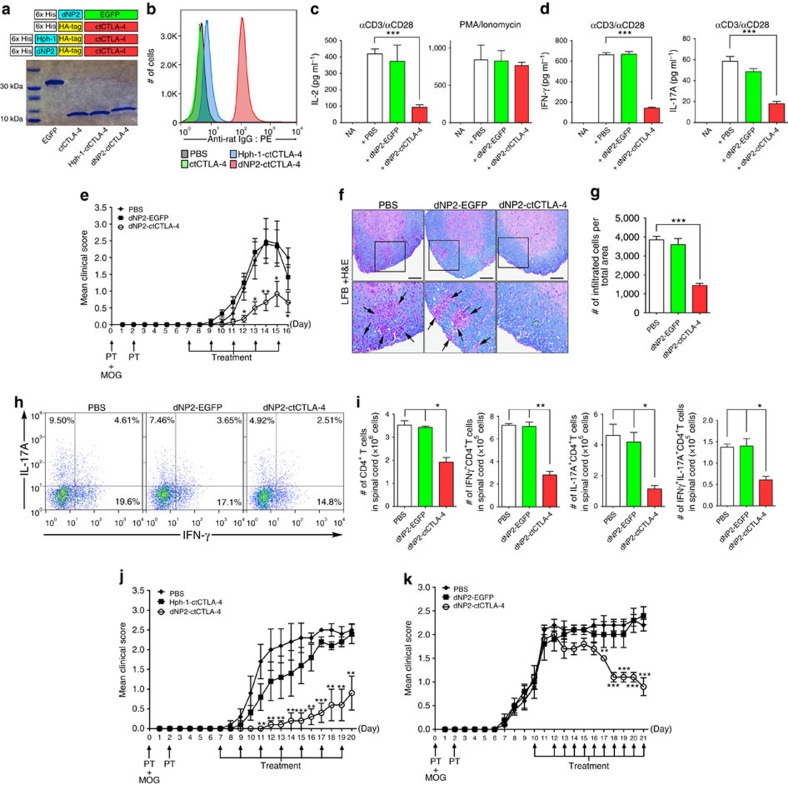
Inhibition of effector T cell functions and amelioration of EAE by dNP2-ctCTLA-4. (**a**) DNA constructs and purified proteins. (**b**) Mouse splenocytes were incubated with 1 μM ctCTLA-4, Hph-1-, and dNP2-ctCTLA-4 for 1 h and the intracellular ctCTLA-4 proteins were stained with an anti-HA antibody and the signal was amplified with PE-conjugated anti-rabbit IgG antibody. Intracellular fluorescence was analysed by flow cytometry. (**c**) Splenocytes from 6-week-old female C57BL/6 mice were activated with anti-CD3/CD28 antibody or PMA/ionomycin in the presence of 1 μM PBS, dNP2-EGFP or dNP2-ctCTLA-4 for 24 h. The concentration of IL-2 was determined by ELISA assay. (**d**) The supernatants of anti-CD3/CD28 antibody stimulated cells were analysed for IFN-γ and IL-17A by ELISA assay. (**e**–**i**) EAE was induced in 7-week-old female C57BL/6 mice as described in the Methods section. The mice were treated intraperitoneally with PBS or 25 μg dNP2-EGFP or dNP2-ctCTLA-4 on day 7 after MOG immunization and subsequently treated every other day (prevention scheme, *n*=15). (**e**) The clinical scores were monitored and (**f**) spinal cord tissues were harvested and observed after Luxol fast blue (LFB) and hematoxylin and eosin staining to determine demyelination and tissue inflammation levels (Scale bar, 100 μm). (**g**) The number of spinal cord tissue infiltrating cells was counted via Image J software. (**h**) The spinal cord cells were isolated and IL-17A and/or IFN-γ expressing CD4 T cells were analysed by flow cytometry. (**i**) Absolute cell numbers in single-cell suspended fractions from the spinal cord were counted and multiplied to determine the proportion of total CD4^+^, IFNγ^+^ CD4^+^, IL-17A^+^CD4^+^ and IFNγ^+^IL-17A^+^CD4^+^ cells. The data are represented as bar graphs (*n*=15). (**j**) EAE was induced as described above. The mice were intraperitoneally treated with 25 μg Hph-1-, dNP2-ctCTLA-4 or PBS every other day from day 7. The clinical scores were monitored every day (*n*=5). (**k**) EAE was induced as described above, then the mice were treated with 100 μg dNP2-EGFP, dNP2-ctCTLA-4 proteins or PBS every day after the clinical score reached 1 (day 10, *n*=5). Values are mean±s.e.m. and *P<0.05; **P<0.01; ***P<0.001; Student's *t*-test.

Using this potent CNS-permeable peptide, dNP2, we generated a DNA construct of dNP2 conjugated to the cytoplasmic domain of CTLA-4, a negative regulator of T-cell co-stimulation^16^,^17^, and purified recombinant protein, as previously described^22^,^23^ (Fig. 5a). We first examined the intracellular delivery efficiency of the recombinant ctCTLA-4 protein in primary mouse CD4 T cells (Fig. 5b). dNP2-ctCTLA-4 exhibited potent intracellular protein delivery compared with Hph-1-ctCTLA-4 at a concentration of 1 μM. Next, we found that dNP2-ctCTLA-4 treatment inhibited IL-2 secretion in splenocytes activated by anti-CD3 and anti-CD28 antibodies, while it had no effect on phorbol 12-myristate 13-acetate and ionomycin stimulation (Fig. 5c). This suggests that the target of dNP2-ctCTLA-4 could be proximal TcR signaling molecules. In addition, IFN-γ and IL-17A expression levels in activated splenocytes were significantly reduced by dNP2-ctCTLA-4 (Fig. 5d). Next, to assess the inhibitory effects of dNP2-ctCTLA-4 in a mouse model of multiple sclerosis, we used C57BL/6 mice immunized with the MOG_35–55_ peptide and treated with pertussis toxin, a standard EAE model. We began intraperitoneal treatment with 25 μg dNP2-ctCTLA-4 on day 7 and continued treating the mice every other day. The clinical scores of the mice treated with dNP2-ctCTLA-4 protein were significantly lower than those of that treated with dNP2-EGFP or phosphate-buffered saline (PBS; Fig. 5e). Then we performed histological analysis of the spinal cord tissue with Luxol fast blue (LFB) and hematoxylin and eosin staining to determine neuronal damage, such as demyelination and tissue inflammation levels. The histological analysis of the spinal cord tissue from dNP2-ctCTLA-4-treated mice showed reduced cellular infiltration and demyelination compared with the other group (Fig. 5f,g). This inhibition of pathological symptoms correlated with a reduced proportion and number of infiltrated IFN-γ- and/or IL-17A-producing CD4 T cells in the spinal cord (Fig. 5h,i). By contrast, 25 μg ctCTLA-4 conjugated with Hph-1, which exhibits less intracellular delivery and brain tissue localization *in vivo* compare with dNP2, did not result in a significant reduction of clinical scores (Fig. 5j). Finally, to examine the therapeutic effects of dNP2-ctCTLA-4 in the EAE model, we intraperitoneally injected 100 μg dNP2-ctCTLA-4 into mice that had already developed the disease and whose average clinical score was 1 (Fig. 5k). Remarkably, dNP2-ctCTLA-4 was able to ameliorate the clinical scores of EAE. Collectively, our data suggest that dNP2-ctCTLA-4 could regulate activated T-cell response *in vitro* and *in vivo* and it could be used as an immune-modulatory protein to control CNS inflammatory diseases such as multiple sclerosis.

## Discussion

In the present study, we identified and optimized a novel CNS-permeable cell membrane-penetrating peptide, dNP2, derived from the human NLBP protein. dNP2 is a tandem repeated form of the NP2 sequence that is highly conserved among various species. dNP2 exhibits potent intracellular protein delivery activity both *in vitro* and *in vivo*. We used *in vitro* and *in vivo* live-cell imaging technologies to directly visualize the dNP2-meditated internalization of macromolecules, such as proteins into T cells or brain cells that are generally regarded to pose huge hurdles for drug delivery. Moreover, we generated a dNP2-conjugated therapeutic protein, using the cytoplasmic domain of CTLA-4, to regulate T-cell responses in an EAE model and observed significant reductions in disease symptoms in both preventive and therapeutic applications.

CPPs have been extensively studied as an approach to deliver macromolecules into the target cells for the last 25 years, ever since the initial elucidation of the cell-permeable ability of the HIV-1 Tat protein^36^. A fusion protein consisting of an 11 amino-acid sequence (TAT) from the Tat protein along with the β-galactosidase protein (TAT-β-gal, 120 kDa) was reported to be delivered into cells, including brain tissue, both *in vitro* and *in vivo*^37^. Various previously characterized CPPs, including Antp^38^, Pep-1 (ref. 39), VP22 (ref. 40) and polyarginine^41^, have also been shown to deliver effector peptides or proteins into cells. CPPs have been widely used to transduce various cargo molecules, including proteins, peptides, small interfering RNA and plasmid DNA, into cells to manipulate cellular responses^42^,^43^,^44^. Recently, polyarginine-conjugated transcription factors, such as Oct4, Sox2, Klf4 and c-Myc, have been used in the generation of induced pluripotent stem cells^45^. Moreover, CPP-conjugated molecules have been studied in clinical trials. Polyarginine-conjugated cyclosporine A was the first clinical-tested CPP-conjugated molecules in phase II trials for the treatment of psoriasis, which was ultimately discontinued in the current^46^. And the TAT-conjugated protein kinase C δ inhibitor (KAI-9803) has completed phase II clinical trials for the treatment of heart attack patients^47^. However, no Food and Drug Administration-approved CPP-based drugs are available, yet implying that the existing CPPs may have less efficiency in human or have side effects such as toxicity. Some previous studies have attempted to improve the intracellular delivery efficiency of TAT using a tandem repeated form of TAT-CPP (dTAT)^48^,^49^. Although dTAT showed much higher delivery efficiency than TAT, we confirmed that dNP2 exhibits much more potent intracellular protein delivery than dTAT in primary T cells *in vitro*, and in various tissues including brain tissue *in vivo* (Supplementary Figs 9 and 10). Moreover, our results demonstrated that dTAT has more significant *in vitro* toxicity than dNP2, suggesting that dNP2 has more advantages over than dTAT for the application purposes.

Previously, the delivery of cargo molecules into brain tissue by TAT peptide was evaluated by indirect methods, such as β-gal enzyme assays^37^ or secondary antibody-based amplification^50^. We, however, visualized the delivered fluorescent protein in the brain tissue of live animals; a direct methodology for tracking protein *in vivo*. We found that only dNP2 delivers proteins into brain cells, including neurons, astrocytes and microglia, through the blood vessels in the brain, while the TAT- and Hph-1-CPP-conjugated proteins mostly remained in the blood vessel. From this, we can deduce that dNP2 may deliver a protein through blood vessels into neurons, astrocytes and microglial cells, which have characteristics to enable uptake of dNP2 proteins. Neuronal cells essentially have endocytosis activity for the recycling of cellular membrane after release of neurotransmitters^51^ and astrocytes and microglial cells are well-characterized phagocytes in the CNS, which have phagocytic activity to clear apoptotic cells or debris^52^. In addition, dNP2-dTomato was significantly co-localized with infiltrated CD4 T cells when the brain inflammation was occurred by MOG_35–55_ immunization, suggesting that dNP2 can deliver its cargo protein not only into the CNS resident cells but also into the infiltrated T cells. We also observed that dNP2 could deliver a protein with high efficiency in human PBMCs, while TAT shows marginal efficiency suggesting that dNP2 may be suitable for clinical purposes in humans.

We also found that among the mixed cell population of PBMCs, phagocytic cells, such as macrophages and dendritic cells, were more likely than lymphocytes to take up CPP-cargo proteins, suggesting that heterogeneity in CPP delivery efficiency among the various cell types. In addition, we observed that the green fluorescent signal present in spleens of dNP2-EGFP-injected mice showed co-localization in mostly F4/80-positive macrophage cells and some of CD4-positive cells, while co-localization in B220-positive cells were not observed (Supplementary Fig. 11); a finding that is similar to results reported for the *in vivo* β-gal delivery by TAT that β-gal activity was more significant in red pulp region^37^.

The mechanisms of cellular uptake of CPPs that have been studied involve lipid raft-mediated endocytosis^35^, clathrin-mediated endocytosis^33^ and macropinocytosis^53^ after interaction with heparan sulphate on cell surfaces^54^. We found that dNP2-recombinant proteins also utilize lipid raft-mediated endocytosis following heparan sulphate interaction, while a short dNP2 peptide alone seems to be able to utilize various endocytic pathways. In addition, we confirmed that the dNP2-EGFP protein could also be more efficiently delivered into human umbilical vascular endothelial cells (HUVEC cells) than TAT-EGFP (Supplementary Fig. 12). This finding suggests that the *in vivo* intracellular localization of a fluorescent protein following intravenous administration could be mediated by interactions with vascular endothelial cells, which might subsequently induce adsorptive-mediated transcytosis in the tissues. The luminal endothelial cell surfaces of the BBB have an overall negative charge with high expression of heparan sulphate proteoglycans^55^. Consequently, possible brain tissue delivery through the BBB could require an initial interaction with heparan sulphate proteoglycan to stimulate endocytosis by endothelial cells, followed by transport into the inner tissue region where astrocytes, neuronal cells and microglial cells accumulate. To understand the potent delivery efficiency of dNP2, we analysed and predicted the three-dimensional (3D) structures of dNP2 using I-TASSER, which is widely used online-based programme^56^,^57^. The predicted 3D-structure of dNP2 possesses two separate α-helical regions linked by a potentially flexible hinge region, whereas the other CPPs contain only a single, linear α-helical region (Supplementary Fig. 13). These structural characteristics may enable increased interactions between dNP2 and heparin sulphate molecules on cells, such as luminal endothelial cells.

To further confirm the brain tissue localization of dNP2 proteins are not mediated by circulating lymphocytes, we demonstrated the brain imaging studies with recombination activating gene 1 knockout (RAG1^−/−^) mice, which have no mature circulating T or B cells, injected with the dNP2-dTomato protein that showed results identical to those of wild-type mice (Supplementary Fig. 14). This suggests that the brain tissue localization of the dNP2-dTomato protein is not dependent on circulating immune cells. Rather, it could be directly absorbed by endothelial cells and then subsequently localized into brain tissue. We attempted various proteins with different molecular weight to evaluate possible size limitations for delivery of proteins into brain tissue. We found that chemically conjugated dNP2-human serum albumin (65 kDa) or dNP2-β-gal (120 kDa) protein could be successfully delivered into brain tissue through blood vessels (Supplementary Fig. 15). Consequently, there seems to be no strict size limitation on transportation into the brain by dNP2, which demonstrate its application potency.

Since the early 1990s, various disease-modifying drugs (DMDs), other than corticosteroids, have been used to treat multiple sclerosis. In 1993, IFN-β 1b, which can reduce pro-inflammatory cytokine production from Th1 cells and inhibit T cell migration into the CNS, was first approved as DMD for multiple sclerosis^58^. GA, a random mixture of polypeptides composed of tyrosine, glutamate, alanine and lysine, can interfere with antigen presentation to T cells by mimicking myelin basic protein^59^. Natalizumab, a monoclonal antibody drug approved for multiple sclerosis in 2004, can bind to α_4_β_1_ molecules on leukocytes, resulting in inhibition of migration of cells into the CNS^60^. Recently, effective and convenient oral drugs, including Fingolimod (2010), Teriflunomide (2012) and dimethyl fumarate (2013), were approved. Fingolimod can modulate the sphingosine 1-phosphate receptor on thymocytes and lymphocytes, and can induce a reduction in inflammatory cell number in the circulation and the CNS^61^. Teriflunomide inhibits the enzyme dihydroorotate dehydrogenase, which plays a critical role in *de novo* synthesis of pyrimidine, and has effects on activated and proliferating T and B cells^62^. Dimethyl fumarate has known protective properties for neurons and modulatory functions in immune responses^63^. Although these DMDs for multiple sclerosis are effective, these drugs have been reported to allow relapse occurrence, have adverse effects on cardiac function^64^, induce possible teratogenicity^62^ and possibly induce progressive multifocal leukoencephalopathy^63^. Because of these issues, significant therapeutic needs remain unmet for multiple sclerosis patients, due to low efficacy in many patients or systemic side effects.

Abatacept consists of the extracellular domain of CTLA-4 and the Fc region of immunoglobulin G. It can bind with B7 molecules on the surface of antigen-presenting cells and inhibit T-cell activation by blocking the co-stimulatory signals to T cells in multiple sclerosis. However, we focused on the importance of the cytoplasmic tail of CTLA-4 in its immune regulatory function, which was previously demonstrated in studies that overexpressed an exon 2-deleted isoform of CTLA-4 (liCTLA-4) in murine T cells^21^ and through mutation of the ligand-binding motif of full-length CTLA-4 (ref. 65), which abolished B7-binding activity. In addition, liCTLA-4 overexpression significantly inhibited the development of type 1 diabetes by regulating T-cell activation and proliferation^66^,^67^. Our results using the cytoplasmic domain of CTLA-4 are in agreement with studies of liCTLA-4, which exhibits immune regulatory functions in transgenic NOD mice independent of B7 binding. We similarly elucidated the function of a B7 independent form of CTLA-4 (ctCTLA-4) that encodes only a part of exon 4 of the *Ctla-4* gene. Other recent studies have demonstrated that 1/4CTLA-4, another isoform of CTLA-4 that genetically resembles ctCTLA-4, enhances T-cell activation and promotes autoimmunity^68^,^69^. However, the alternative splicing of 1/4CTLA-4 induces frame-shifts that produce a completely different amino acid sequence than ctCTLA-4 (refs 69, 70). dNP2-ctCTLA-4 inhibited T-cell activation and displayed potent therapeutic effects in an autoimmune encephalomyelitis model, suggesting that the levels of the CTLA-4 cytoplasmic domain in T cells are important for its function. Although dNP2-ctCTLA-4 could be delivered into macrophages and dendritic cells, there was no significant alteration of inflammatory cytokine production by Toll-like receptor ligand stimulation in those cells, while it could successfully inhibit IL-2 or IFN-γ production by activated CD4 and CD8 T cells (Supplementary Fig. 16). This suggests that the inhibitory mechanism on EAE is not mediated by suppression of innate immune cells, but rather through inhibiting effector T-cell functions. Importantly, our *in vivo* experiments demonstrate that dNP2-ctCTLA-4 treatment ameliorated clinical symptoms, even when administration was initiated after tail paralysis. This suggests that the inhibition of effector T-cell functions by dNP2-ctCTLA-4 could modulate the progression of disease symptoms or enhances recovery without significant *in vivo* toxicity (Supplementary Fig. 7).

Taken together, our study clearly demonstrates that a novel and potent CNS-permeable peptide, dNP2, can deliver macromolecules, such as proteins, into the brain and spinal cord. New therapies based on the use of dNP2 to deliver ctCTLA-4 into CNS-infiltrating T cells could be effective in regulating multiple sclerosis. Future studies will focus on optimizing the functional sequence of dNP2-ctCTLA-4 to increase its efficiency and stability.

## Methods

### Purification of recombinant proteins

CPP-conjugated fluorescent proteins were purified using bacterial systems. *Escherichia coli* BL21(DE3) Star pLysS were transformed with pRSET-b plasmids encoding CPP-conjugated fluorescent proteins and incubated in 50 ml of Luria-Bertani broth containing ampicillin for 12 h at 37 °C. The cultures were then transferred into 500 ml of fresh Luria-Bertani medium and incubated at 37 °C for another 1–2 h until the optical density at 600 nm (OD_600 nm_) reached 0.4–0.6, after which 1 mM isopropyl-β-D-thiogalactopyranoside was added. The cells were subsequently incubated overnight at 20 °C with shaking at 150 r.p.m. (for fluorescent proteins) or for 5 h at 37 °C and 200 r.p.m. (for ctCTLA-4 proteins). The cells were then harvested by ultra-centrifugation at 6,000 r.p.m. for 20 min at 4 °C and resuspended in native lysis buffer (10 mM imidazole, 300 mM NaCl, 50 mM NaH_2_PO_4_, pH 8.0) or 6 M urea lysis buffer and sonicated using a Vibra-cell VCX-130 ultrasonic processor (Sonics and Materials Inc., Newton, CT). The supernatants were filtered through 0.45-μm cellulose nitrate syringe filters. The 6-His-tagged proteins were purified via Ni-NTA affinity chromatography (Qiagen) and desalted using a PD-10 Sephadex G-25 column (GE Healthcare). To obtain highly purified proteins, an additional ion-exchange protein purification step was performed using SP Sepharose High Performance (GE Healthcare), followed again by desalting on a PD-10 Sephadex G-25 column. Protein concentrations were measured using the Bradford assay.

### Cell lines and cell culture

Jurkat (human leukaemia cells) cells were purchased from the American Type Culture Collection (ATCC) and maintained in Roswell Park Memorial Institute (RPMI) 1640 media supplemented with 10% fetal bovine serum (FBS) and 1% penicillin/streptomycin antibiotics. HeLa (human cervix epithelial carcinoma cells) cells were purchased from the ATCC and cultured in Dulbecco's modified Eagle's media with GlutaMAX supplemented with 10% FBS and 1% penicillin/streptomycin antibiotics. All cells were maintained at 37 °C in a 5% CO_2_ incubator. All of the above reagents were purchased from Thermo Scientific Hyclone.

### Mice

Male or female C57BL/6 mice, 6–8 weeks old, were obtained from Orient Bio (Daejeon, Korea). The mice were housed and maintained in a specific pathogen-free facility at Hanyang University under controlled conditions with constant temperature (21±1 °C), humidity (50±5%), and a 12 h light/dark cycle with regular chow and autoclaved water. All animal protocols used in this study were approved by the Hanyang Animal Care and Use Committee.

### *In vitro* delivery efficiency of dNP2

Jurkat T cells were cultured in 24-well plates at 5.0 × 10^5^ cells per well in RPMI 1640 media. After the cells were seeded, each protein was added for the indicated times. Following incubation, the cells were harvested and washed three times with PBS. Intracellular fluorescence was analysed on a fluorescence-activated cell sorting (FACS) Canto II flow cytometer (BD Bioscience), and the data were analysed using FlowJo software (Tree Star, Inc.). The spleens from 6-week-old female C57BL/6 mice were placed in 60 × 15 mm cell culture dishes containing 3 ml of PBS. Single-cell suspensions were physically generated using a 0.45-μm pore-cell strainer and centrifuged after the addition of 10 ml fresh PBS. Red blood cells were lysed in Ammonium-Chloride-Potassium (ACK) buffer (0.15 M NH_4_Cl, 10 mM KHCO_3_, 1 mM EDTA-2Na, pH 7.2). Of all, 1.0 × 10^6^ splenocytes per well were seeded and the delivery efficiencies of CPP-proteins were analysed. The cells were subdivided into various cell types by staining with 1/800 diluted anti-mouse CD4-PerCP-Cy5.5 (#45-0042) and anti-mouse CD19-PE-Cy7 (#25-0193) or anti-mouse F4/80 PerCP-Cy5.5 (#45-4,801), anti-mouse MHCII-PE (#12-5,322-81), anti-mouse CD11b-PE-Cy7 (#25-0112) and anti-mouse CD11c-APC (#17-0114) FACS antibodies. The antibodies were purchased from eBioscience.

### *In vitro* toxicity assay

The viability of the cells was measured via water-soluble tetrazolium-8 based cell counting kit-8 (CCK-8, Dojindo). A total of 5.0 × 10^3^ HeLa cells were seeded in 96-well plate and then treated with 10, 30, 50 or 100 μM TAT-, dTAT-, NP2-, dNP2-EGFP or PBS for 24 h. After incubation, the cells were washed with PBS and further incubated with CCK-8 solution for 2 h. The optical density values were then analysed by plate reader at 450 nm (Bio-Rad).

### Intracellular localization of dNP2

HeLa cells were seeded in six-well plates at 1.0 × 10^6^ cells per well in Dulbecco's modified Eagle's media and incubated overnight. The HeLa cells were then treated with CPP-EGFPs or -dTomatos (20 μM) for 2 h at 37 °C. Following incubation, the medium was exchanged, and the cells were washed three times with PBS. The cells were fixed in 4% paraformaldehyde and washed three times with PBS. Hoechst 33342 (Invitrogen) was also added to stain nuclei, followed by three washes with PBS. Cells were then mounted with mounting media (Sigma-Aldrich). The intracellular localization of EGFP fluorescence was observed using a DeltaVision imaging system (Applied Precision) and the intracellular localization of dTomato fluorescence was observed using a confocal microscope (Nikon Instruments Inc.).

### Isolation of human PBMC and *in vitro* delivery of dNP2

The protocol described here was approved by the Institutional Review Board of Hanyang University. Human blood samples were obtained from healthy donors and blood lymphocytes were isolated by density centrifugation using Ficoll-Paque PLUS (GE Healthcare). The isolated lymphocytes were seeded 1.0 × 10^6^ cells per well and the delivery efficiencies of CPP-proteins were analysed. The cells were further stained with 1/800 diluted anti-human CD4-PE-Cy7 (#25-0049), anti-human CD19-APC (#17-0199), anti-human CD11b-PE-Cy7 (#25-0118) or anti-human CD11c-APC (#17-0116) FACS antibodies purchased from eBioscience.

### Live imaging of primary CD4^+^ T cells

6-week-old female C57BL/6 mice were euthanized, and CD4^+^ T cells were isolated from spleens and lymph nodes using a CD4^+^ T cell negative selection kit (StemCell Technologies, INC). Isolated CD4^+^ T cells in RPMI media were seeded on anti-CD44 antibody-coated glass coverslips mounted in Chamlide chambers. The protein solution was then added to the chamber, and time-lapse imaging was initiated. Differential interference contrast (DIC) and GFP images were recorded at 5-min intervals for 2 h. The acquired time-lapse images were analysed using MetaMorph or ImageJ software 1.48v.

### Delivery mechanisms of dNP2

Isolated splenocytes from 6-week-old female C57BL/6 mice were incubated in the presence of CPP proteins or peptides for 1 h at various temperatures (4, 25 or 37 °C). The splenocytes or HeLa cells were pre-treated with heparin (0, 10, 20 or 50 μg ml^−1^), MβCD (0, 3, or 5 mM), chlorpromazine (0, 10 or 30 μM) or amiloride (0, 1, 2 or 5 mM) for 30 min at 37 °C, before being treated with CPP-proteins or -peptides and additionally incubated with CPP proteins or peptides for 1 h at 37 °C. All cells were trypsinized (trypsin, Thermo Scientific Hyclone) and washed with FACS buffer (PBS containing 10% FBS, 5% sodium azide and 1% EDTA). Heparin, MβCD, chlorpromazine and amiloride were purchased from Sigma-Aldrich.

### Live imaging using multi-photon microscopy

For *in vivo* multi-photon imaging of the brain, 8-week-old male C57BL/6 mice (23–25 g) were subjected to surgery to introduce an observation window in the skull. Animals were anaesthetised via isoflurane inhalation and maintained at body temperature (37–38 °C) using a homoeothermic heating pad system controlled by a rectal probe. Isoflurane levels were set at 3% to induce anaesthesia and were maintained at 1.5% during cranial window surgery or multi-photon imaging. The animals were closely monitored throughout the entire procedure to ensure physiological health. All surgical procedures were approved by the Institutional Animal Care and Use Committee (IACUC) of Sungkyunkwan University. Animals were fixed in a stereotaxic frame (David Kopf Instruments, Tujunga, CA), and a round cranial window with a diameter of 3 mm was created on the right hemisphere, centred at ML, +2.5 mm, AP, −1.5 mm. After craniotomy, a customized chamber plate (Narishige Inc., Tokyo, Japan) with a 5-mm observation hole was placed on the opened craniotomy area and fixed with dental resin. The cranial window was then filled with sterilized artificial cerebrospinal fluid (125 mM NaCl, 2.5 mM KCl, 25 mM NaHCO_3_, 1.25 mM NaH_2_PO_4_, 2 mM CaCl_2_, 1 mM MgSO_4_, 10 mM glucose, pH 7.4) and covered with a 7-mm coverslip. The cranial window was sealed with cyanoacrylic glue, and the animals were then placed in a head-fixing apparatus (MAG-1, Narishige Inc.) for multi-photon microscopy (TCS SP8 MP, Leica Microsystems CMS GmbH). Excitation was performed using a 900-nm Ti:Sapphire laser (Chameleon Vision II, Coherent Inc.), and the emitted fluorescent signal was detected on a hybrid detector through a 585/40 band-pass filter cube. To track the delivery of the carrier peptide into the brain tissue, 3D z-stack images were obtained with a 20-min time interval for 2 h following the injection of the carrier peptide via the tail vein (2.5 mg per animal). The imaged brain size was 354.29 × 354.29 μm^2^ (1024 × 1024 pixels) and was acquired using a 25 × water-immersion objective lens (N.A. 0.95). The imaging depth was approximately 450–500 μm from the brain surface, with a resolution of 1 μm. Following acquisition, the images were analysed using LAS AF 3.2.0 (Leica Microsystems CMS GmbH) and Imaris 7.7.2 (Bitplane) software.

### Immunofluorescence

6-week-old male C57BL/6 mice were intraperitoneally injected with 5 mg of dNP2-dTomato or control CPP-dTomato proteins to analyze the systemic delivery efficiency of dNP2-dTomato. The mice were killed 2 h after injection. The tissues were then harvested, washed with PBS and fixed with 4% paraformaldehyde. All harvested tissues were frozen using O.C.T. compound (WAKO Chemical). The frozen blocks were cut into 6-μm-thick slices using a cryostat (Thermo Scientific) and examined via fluorescence microscopy (Leica Microsystems). For the analysis of brain and spinal cord tissues in EAE-induced mice, 1 h after intravenous injection of 2.5 mg dNP2-dTomato, the animals were euthanized and perfused transcardially with 15 ml of PBS, followed by 15 ml of 4% paraformaldehyde. The brain tissue was removed and washed with PBS. The tissue was then post-fixed in 4% paraformaldehyde for 1 h at room temperature and cryoprotected in 30% sucrose for 24 h at 4 °C. Brain tissues were frozen using O.C.T. compound (WAKO Chemical). The frozen blocks were cut into 40-μm-thick slices using a cryostat (Thermo Scientific, Logan, UT). Sections were incubated in cold acetone for 30 min at −20 °C and then washed with PBS for 30 min at room temperature. Washed samples were incubated with permeabilization buffer (0.5% Triton X-100 in PBS) for 10 min and blocking buffer (3% BSA, 0.1% Tween-20) for another 20 min. Primary antibody staining was performed overnight using 1/100 diluted anti-mouse CD4-FITC (eBioscience, #11-0041), 1/100 diluted anti-mouse GFAP (Millipore, #AB5804), 1/100 diluted anti-mouse Iba-1 (WAKO chemical, #019-19741) or 1/100 diluted anti-mouse NeuN (Abcam, #104224). Nuclei were stained with Hoechst 33342 dye (Invitrogen) or DAPI (Vector Laboratory) following antibody binding. All section samples were analysed by confocal microscopy (TCS SP8, Leica Microsystems CMS GmbH).

### EAE induction

7-week-old female C57BL/6 mice were purchased from DBL. The protocol described here was approved by the animal experimentation ethics committee of Hanyang University. EAE was induced by subcutaneous immunization with 100 μg of MOG_35–55_ peptide (MEVGWYRSPFSRVVHLYRNGK) in Freund's adjuvant emulsion (adjuvant-incomplete Freund and *Mycobacterium tuberculosis* H37Ra at 4 mg per ml, BD Difco). The total subcutaneously injected emulsion volume was 200 μl. At 0 h and 48 h after immunization, mice were intraperitoneally treated with 200 ng of pertussis toxin (List Biological Laboratories Inc.). Animals were scored daily for signs of clinical disease. Each dose of protein diluted in 100 μl of PBS or fresh PBS alone was injected intraperitoneally. Mice were euthanized at the end of the experiments, and lymphocytes in the CNS were isolated by Percoll (GE Healthcare) density-gradient centrifugation. The surface of the isolated lymphocytes was stained with 1/800 diluted anti-mouse CD4 PerCP-Cy5.5 antibody (eBioscience, #45-0042). The lymphocytes were also stained with 1/400 diluted anti-mouse IFN-*γ*-FITC (eBioscience, #13-7,311) and 1/100 diluted IL-17A-APC antibodies (eBioscience, #17-7,177) using the Fixation/Permeabilization concentrate and diluent kit (eBioscience). The cells were analysed using a FACSCanto flow cytometer and FlowJo software. For histologic analysis, paraffin blocks of spinal cord tissues were deparaffinized and immersed in Luxol fast blue. For combination staining, hematoxylin and eosin was used (Dako). The infiltrated cells in the white matter region of spinal cord tissues were counted using Image J software 1.48v.

### *In vivo* toxicity assays

Three groups of 7-week-old female C57BL/6 mice were repetitively injected with PBS, 5 mg per kg of dNP2-dTomato or dNP2-ctCTLA-4, respectively, every other day for 14 days. The weight changes of the mice were daily monitored. At day 15, the mice were sacrificed and the morphologies of the spleen, liver and brain were carefully observed. The cytotoxicity of each protein against splenocytes and thymocytes was analysed using an Annexin V and 7-AAD staining kit (BD bioscience). The percentages of naive CD4 T cells in lymph nodes and spleen were analysed with isolated lymphocytes from each tissue after staining with 1/800 diluted anti-mouse CD4-PerCP-Cy5.5 (#45-0042), anti-mouse CD62L-FITC (#11-0621) and anti-mouse CD44-PE (#12-0441) FACS antibodies purchased from eBioscience. Liver toxicity of the proteins was analysed using an alanine aminotransferase activity assay kit (BioVision) and an aspartate aminotransferase activity assay kit (BioVision).

### Statistics

The data were analysed using two-tailed Student's *t*-tests. *P* values <0.05 were considered significant.

## Additional information

**How to cite this article:** Lim, S. *et al.* dNP2 is a blood–brain barrier-permeable peptide enabling ctCTLA-4 protein delivery to ameliorate experimental autoimmune encephalomyelitis. *Nat. Commun.* 6:8244 doi: 10.1038/ncomms9244 (2015).

## Supplementary Material

Supplementary Information Supplementary Figures 1-16
