# Supplementary material for: dNP2 is a blood–brain barrier-permeable peptide enabling ctCTLA-4 protein delivery to ameliorate experimental autoimmune encephalomyelitis
Source: Nat Commun. 2015 Sep 15;6:8244. doi: 10.1038/ncomms9244 (PMC4579786; doi:10.1038/ncomms9244)
Supplement: Supplementary Information — Supplementary Figures 1-16 [file ncomms9244-s1.pdf]

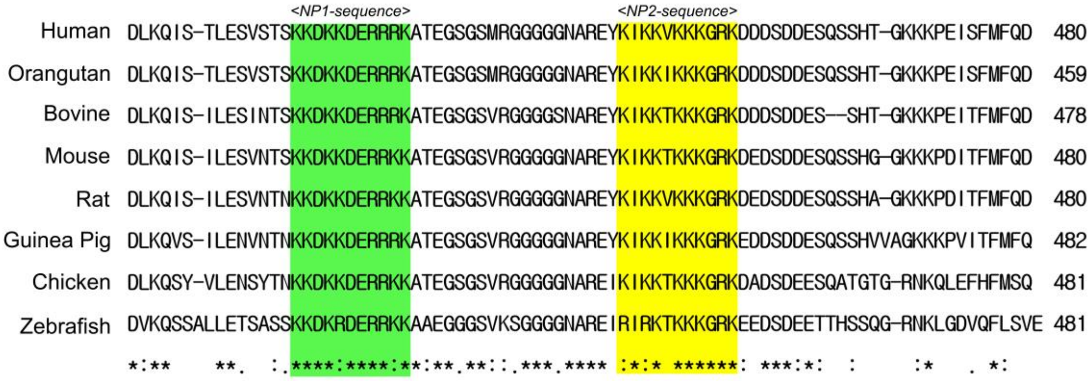

Supplementary Figure 1. NP1- and NP2-sequences are highly conserved among species. The sequences of E3 UFM1-protein ligase 1 (in human, also known as novel LZAP-binding protein, NLBP) from various species were aligned using the *ClustalW2 multiple sequence alignment program from EMBL-EBI*. The green box indicates the conserved region of the NP1-sequence among the various species and the yellow box indicates the conserved region of the NP2-sequence. ":" indicates the amino acids with the same properties. "*" indicates the same amino acids. Blank indicates different properties.

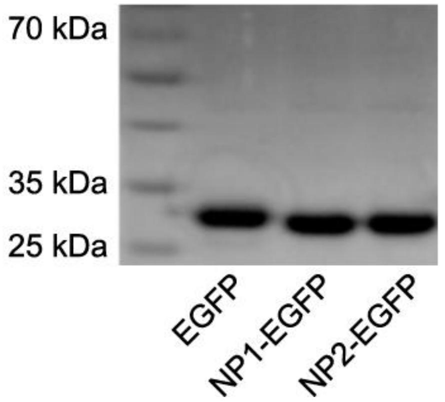

Supplementary Figure 2. Purified EGFP, NP1-EGFP and NP2-EGFP proteins. Ni-NTA column-purified EGFP, NP1-EGFP and NP2-EGFP proteins were analyzed by SDS-PAGE.

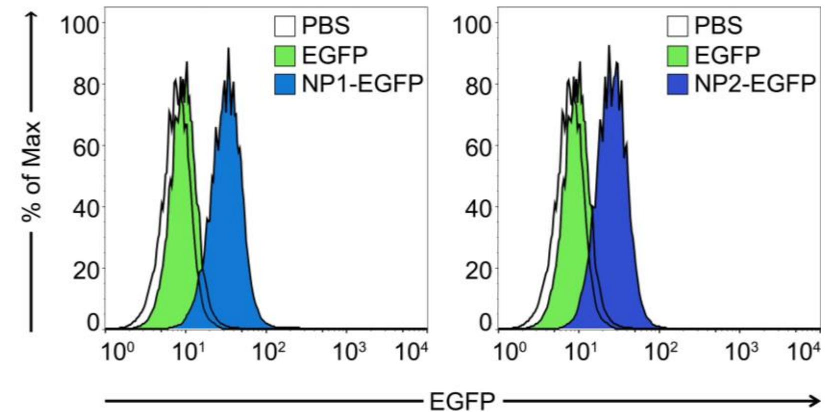

Supplementary Figure 3. Intracellular protein delivery efficiencies of NP1- and NP2-EGFP in Jurkat T cells. Jurkat T cells were incubated with 5 μM EGFP, NP1-EGFP, NP2-EGFP or PBS for 2 h at 37°C and intracellular fluorescence was analyzed by flow cytometry.

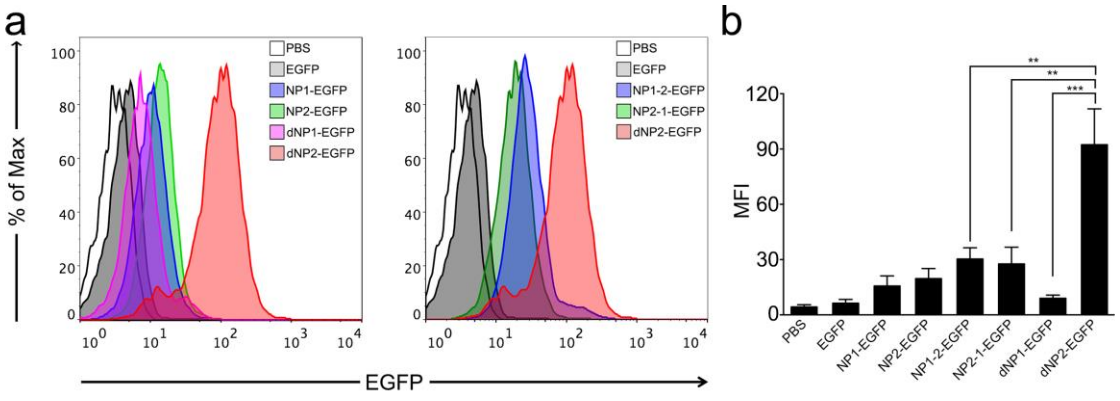

Supplementary Figure 4. Comparative analysis of the intracellular protein delivery efficiency using combinations of NP-1 and NP-2 sequence in Jurkat T cells. (a) Jurkat T cells were incubated with 5 μM of EGFP, NP1-, NP2-, dNP1-, dNP2-, NP1-2-, NP2-1-EGFP or PBS for 2 h. The intracellular fluorescence was analyzed by flow cytometry. (b) The results were indicated as mean fluorescence intensity (MFI) of the cells. Values are mean±s.e.m. and ** indicates $p<0.01$; *** indicates $p<0.001$; Student's *t*-test.

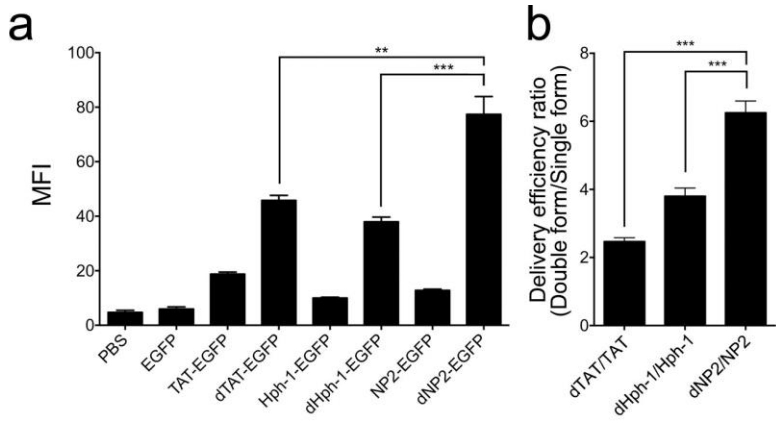

Supplementary Figure 5. Comparative analysis of the intracellular protein delivery efficiency of monomer and tandem-repeated form using various CPPs. Jurkat T cells were incubated with 5 μM EGFP, TAT-, dTAT-, Hph-1-, dHph-1-, NP2-, dNP2-EGFP or PBS for 2 h. (a) Intracellular fluorescence was analyzed by flow cytometry and represented as mean fluorescence intensity (MFI) of the cells. (b) The delivery efficiency ratio of double forms/single forms were calculated and represented as a bar graph. Values are mean±s.e.m. and ** indicates $p<0.01$; *** indicates $p<0.001$; Student’s *t*-test.

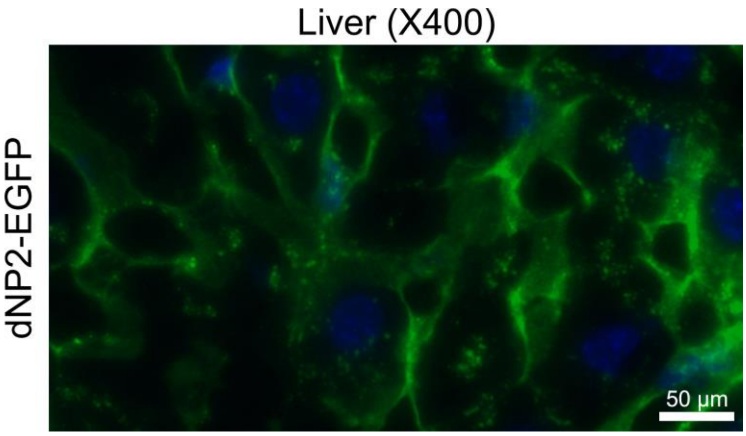

Supplementary Figure 6. Intracellular localization of dNP2-EGFP in the liver tissue. A C57BL/6 mouse was injected intraperitoneally with 5 mg dNP2-EGFP. Two hours after injection, the liver of the mouse was observed at high magnification to determine the localization of dNP2-EGFP in the tissue. Blue regions indicate nuclei, which were stained with Hoechst and the green dots and regions indicate EGFP (X400, scale bar = 50 μm).

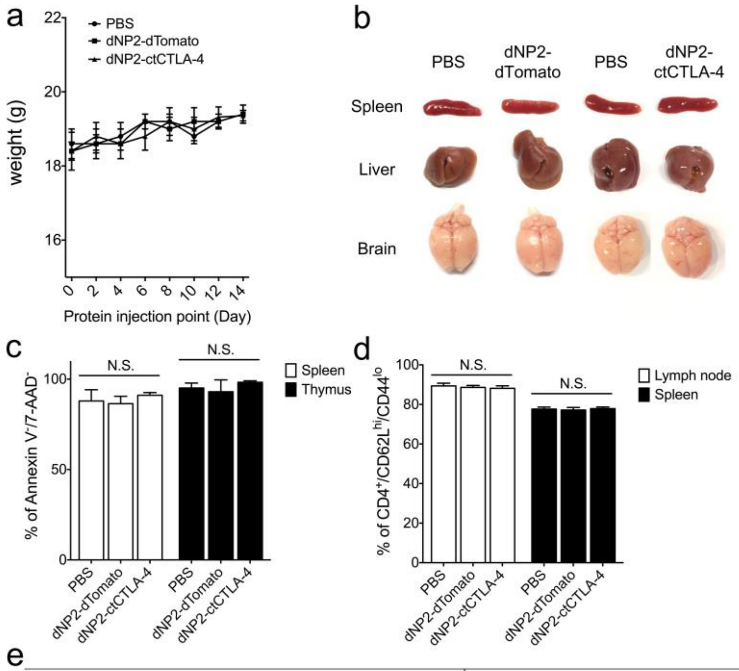

e

	ALT activity		AST activity	
	Mean	SEM	Mean	SEM
PBS	6.9979	± 1.8851	17.6155	± 1.8851
dNP2-dTomato	5.7666	± 2.6004	14.5566	± 2.6004
dNP2-ctCTLA-4	6.0312	± 2.9666	17.1887	± 2.9666

n=5 in each group. The results does not have statistically significance.

Supplementary Figure 7. No significant *in vivo* toxicity of dNP2-recombinant proteins. Groups of mice were repetitively treated with PBS, dNP2-dTomato (5 mg per kg) or dNP2-ctCTLA-4 (5 mg per kg) every other day for 14 days. (a) Body weight was measured every other day and no significant changes were observed. (b) The spleens, livers and brains were harvested, observed and no significant abnormalities were seen. (c) Splenocytes and thymocytes were isolated and the proportion of live cells was analyzed using an Annexin V/7-AAD staining kit. No significant abnormalities were noted. (d) The proportions of naïve CD4 T cells ($CD4^{+}CD62L^{+}CD44^{lo}$) in inguinal lymph node and spleen, were analyzed by flow cytometry. No significant abnormalities were found. (e) No significant abnormalities in systemic *in vivo* liver toxicity were observed using an alanine aminotransferase (ALT) activity assay kit and an aspartate aminotransferase (AST) activity assay kit. Values are mean±s.e.m. and N.S. indicates non-significant; Student's *t*-test.

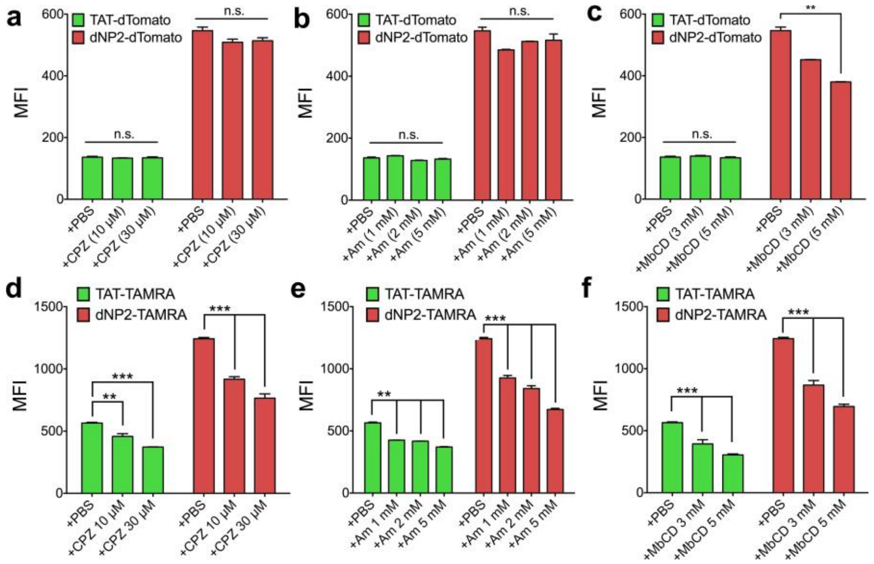

Supplementary Figure 8. Intracellular protein delivery mechanisms of dNP2-peptide and dNP2-protein in total splenocytes. Splenocytes were pre-treated with the indicated concentrations of chlorpromazine (CPZ), amiloride (Am) or methyl-beta-cyclodextrin (MbCD) for 30 min at 37°C. (a-c) The cells were further incubated with 5 μM TAT- or dNP2-dTomato protein for 1 h at 37°C or (d-f) incubated with 5 μM TAMRA-labeled TAT or dNP2 peptide for 1 h at 37°C. The cells were analyzed with flow cytometry and the data are represented as mean fluorescence intensity (MFI) of the total splenocytes. Values are mean±s.e.m. and ** indicates $p<0.01$; *** indicates $p<0.001$; N.S. indicates non-significant; Student's *t*-test.

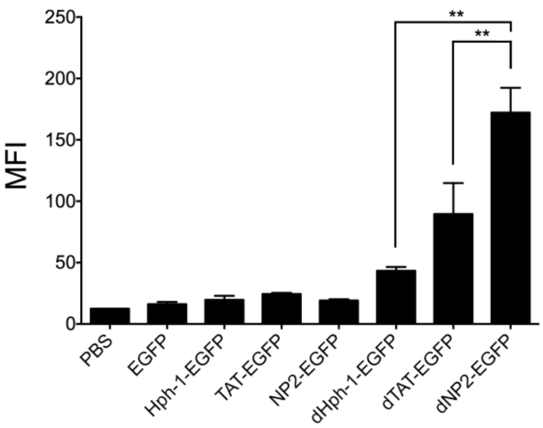

Supplementary Figure 9. Comparative analysis of the intracellular protein delivery efficiencies of tandem-repeated CPPs in splenocytes. Splenocytes were incubated with 5 μM EGFP, Hph-1-, TAT-, NP2-, dHph-1-, dTAT-, dNP2-EGFP or PBS for 2 h. Intracellular fluorescence was analyzed by flow cytometry and the data are represented as mean fluorescence intensity (MFI) of the cells. Values are mean±s.e.m. and ** indicates $p<0.01$; Student's *t*-test.

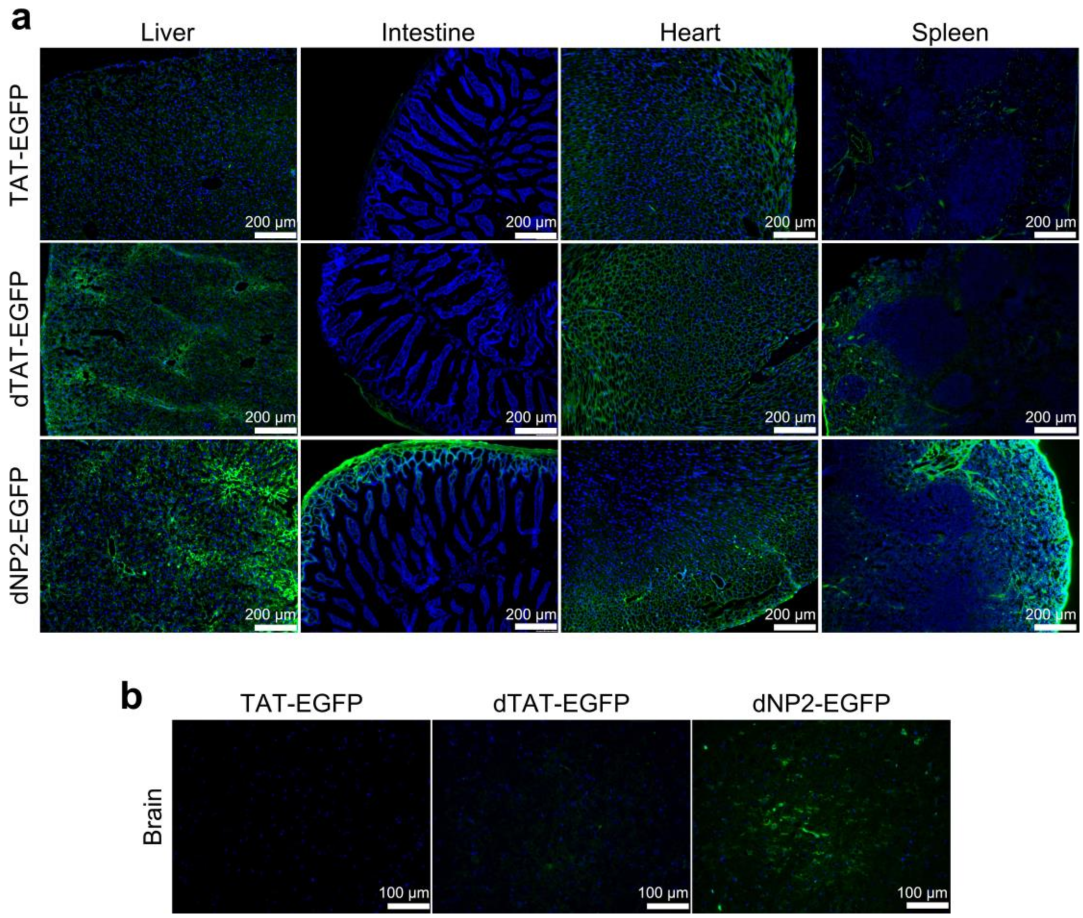

Supplementary Figure 10. Comparison of the protein delivery efficiencies of dNP2 and dTAT in mice. (a) 6-week-old C57BL/6 mice were intraperitoneally injected with 5 mg TAT-EGFP, dTAT-EGFP or dNP2-EGFP. Two hours after injection, liver, intestine, heart and spleen tissues were harvested and the EGFP signal from the frozen sectioned tissues was observed by fluorescence microscopy (X100, scale bar = 200 μm). (b) The brain tissues were harvested and the EGFP signal from the frozen sectioned brain slide was observed by fluorescence microscopy (X200, scale bar = 100 μm).

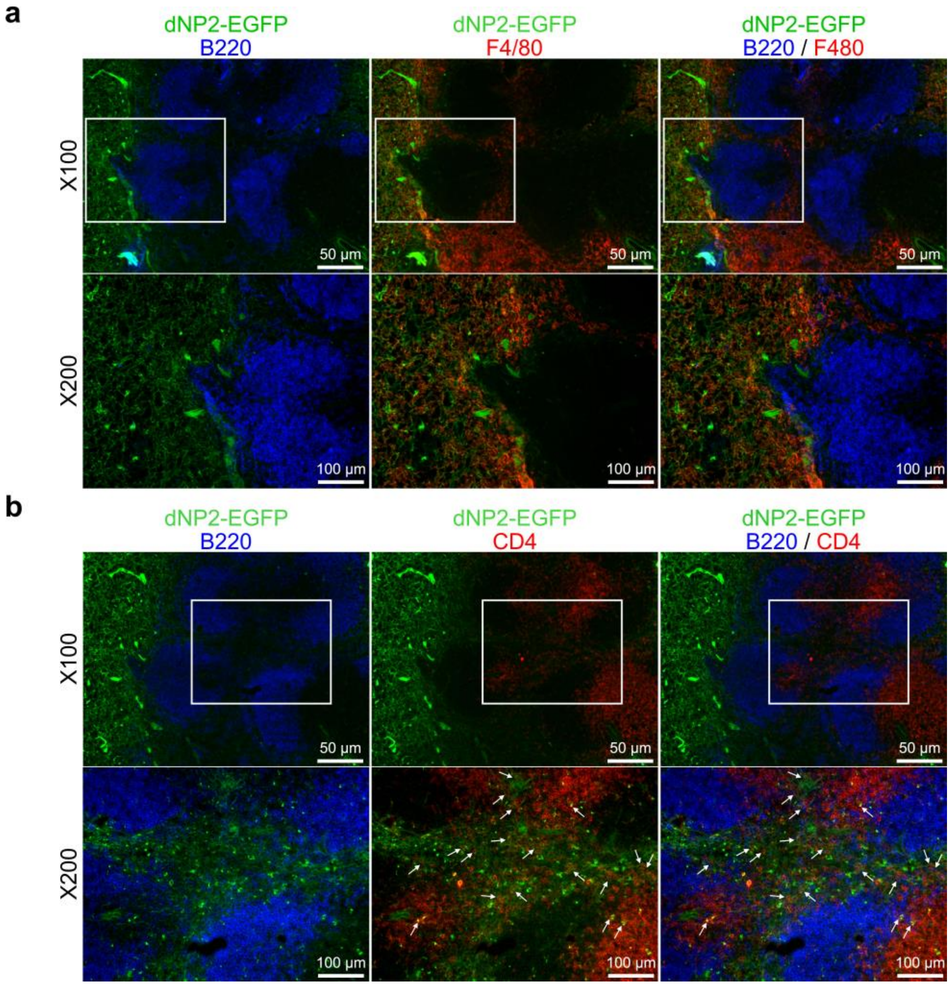

Supplementary Figure 11. Cell type preference of intracellular protein delivery of dNP2-EGFP in mouse spleen. A 6-week-old C57BL/6 mouse was intraperitoneally injected with 5 mg dNP2-EGFP. Two hours after injection, the spleen was harvested and (a) the EGFP signal (green) in macrophages (anti-F4/80-PE, red) and B cells (anti-B220-APC, blue) or (b) the EGFP (green) signal in CD4 T cells (anti-CD4-PE, red) and B cells (anti-B220-APC, blue) from the frozen sectioned slide was observed by fluorescent microscopy (X100; scale bar = 50 µm., X200; scale bar = 100 µm).

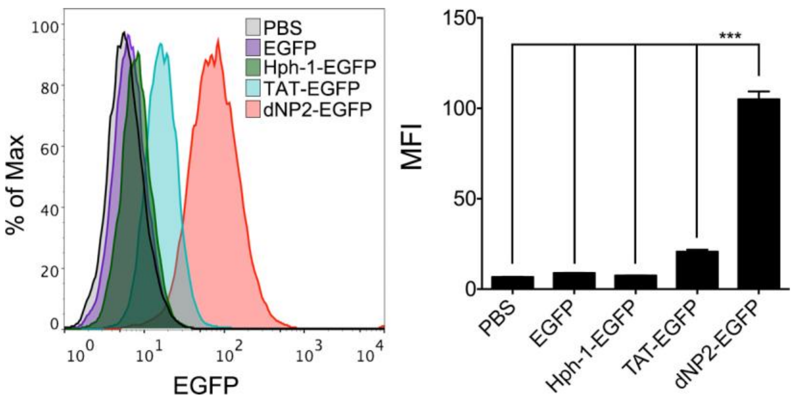

Supplementary Figure 12. Protein delivery efficiency of dNP2 into human umbilical vascular endothelial cells (HUVEC). HUVEC cells were incubated in the presence of 5 μM EGFP, Hph-1-EGFP, TAT-EGFP, dNP2-EGFP or PBS for 2 h at 37°C. Intracellular fluorescence was analyzed by flow cytometry and the data was represented as histogram and mean fluorescence intensity (MFI). Values are mean±s.e.m. and *** indicates $p<0.001$; Student's *t*-test.

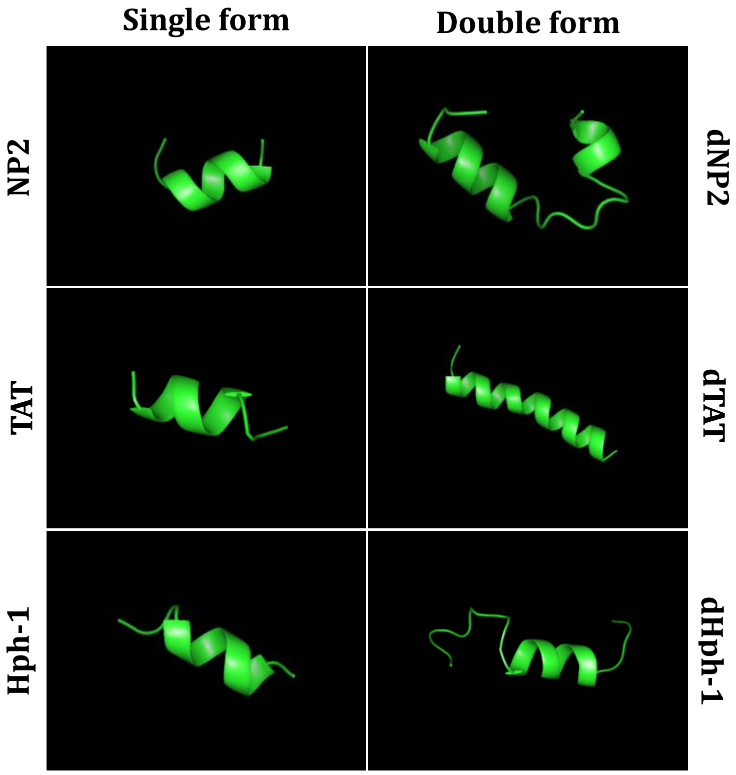

Supplementary Figure 13. Predicted 3D-structures of CPPs. (a) 3D-structures of each CPPs were predicted by I-TASSER online. The data were represented using Jmol (an open-source Java viewer for chemical structure in 3D. http:www.jmol.org).

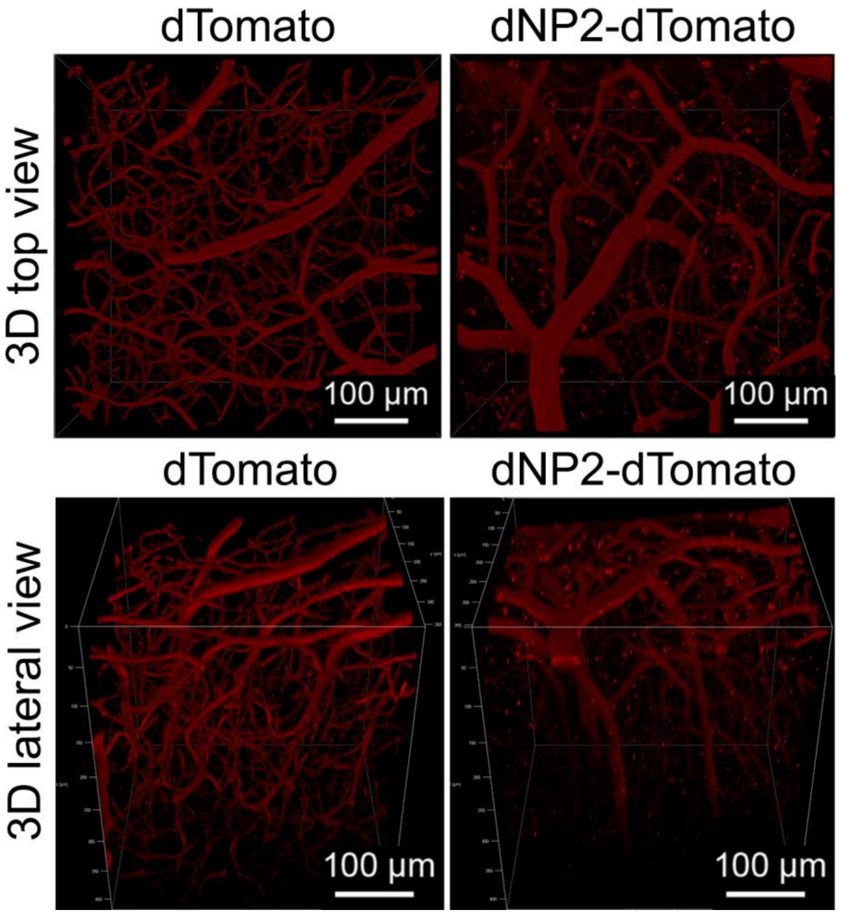

Supplementary Figure 14. dNP2 delivers a protein into the brain not via peripheral circulating lymphocytes. 2.5 mg dTomato or dNP2-dTomato proteins were intravenously injected into RAG1$^{-/-}$ mice through the tail vein after anesthesia to allow for real time live confocal microscopic analysis. Mouse brains were observed 100 min after injection via multi-photon confocal microscopy using a 3D lateral view (X300, scale bar = 100 μm)

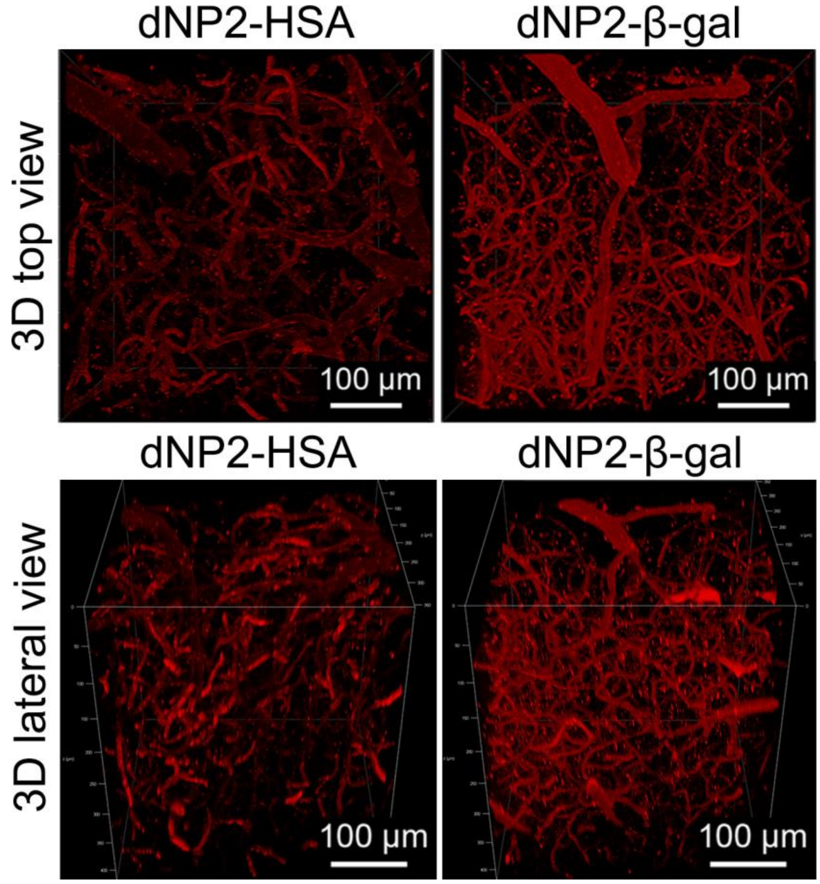

Supplementary Figure 15. dNP2 delivers various size **protein cargos into the brain.** 0.5 mg dNP2-TAMRA conjugated human serum albumin (HSA, 65 kDa) and *β*-galactosidase (*β*-gal, 120 kDa) proteins were intravenously injected into wild type C57BL/6 mice through the tail vein after anesthesia to allow for real time live confocal microscopic analysis. Mouse brains were observed 100 min after injection via multi−photon confocal microscopy using a 3D lateral view (X300, scale bar = 100 μm)

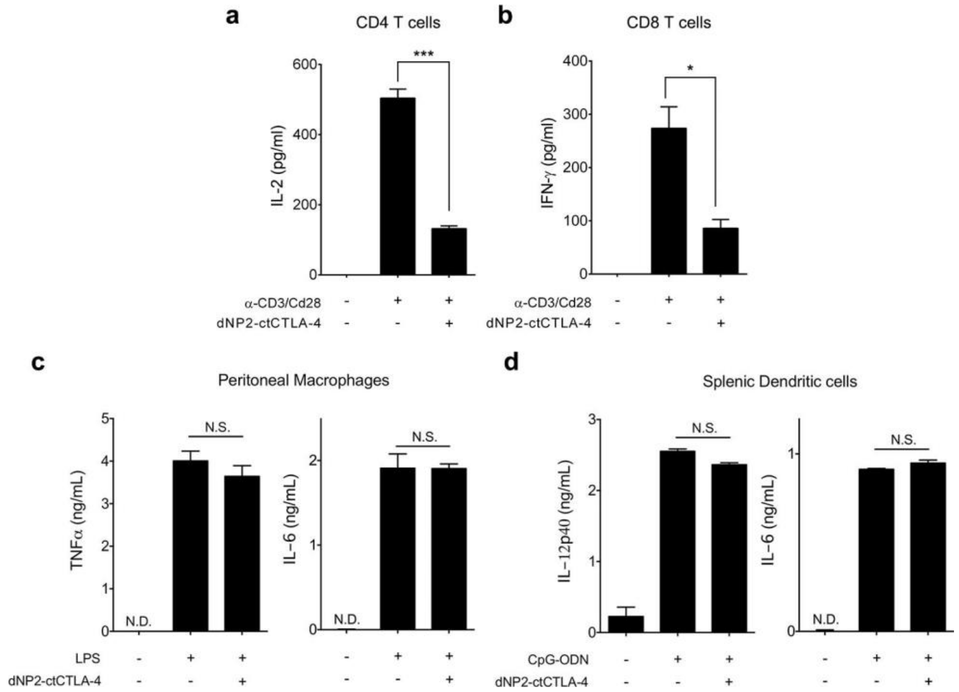

Supplementary Figure 16. dNP2-ctCTLA-4 specifically regulates activated T cell functions. (a, b) MACS-purified splenic naïve CD4 T cells ($CD4^{+}CD25^{-}CD6L^{+}$) or FACS-sorted splenic CD8 T cells were activated with plate-bound 2 μg per ml of anti-CD3 and anti-CD28 antibodies. (a) IL-2 production of activated CD4 T cells was analyzed by ELISA assay. (b) IFN-γ production of activated CD8 T cells was analyzed by ELISA assay. (c) Peritoneal macrophages were isolated from the peritoneal cavities of 6-week-old C57BL/6 mice and the cells were activated with 10 ng per ml lipopolysaccharide with or without 1 μM dNP2-ctCTLA-4. TNFα and IL-6 production were analyzed by ELISA assay. (d) FACS-sorted splenic dendritic cells ($CD11c^{+}$) were activated with 5 μg per ml CpG-ODN with or without 1 μM dNP2-ctCTLA-4. IL-12p40 and IL-6 production were analyzed by ELISA. Values are mean±s.e.m. and N.D. indicates non-detectable; N.S. indicates non-significant; Student's *t*-test.
